# Shifting Distributions of Adult Atlantic Sturgeon Amidst Post-Industrialization and Future Impacts in the Delaware River: a Maximum Entropy Approach

**DOI:** 10.1371/journal.pone.0081321

**Published:** 2013-11-08

**Authors:** Matthew W. Breece, Matthew J. Oliver, Megan A. Cimino, Dewayne A. Fox

**Affiliations:** 1 University of Delaware, Lewes, Delaware, United States of America; 2 Delaware State University, Dover, Delaware, United States of America; North Carolina State University, United States of America

## Abstract

Atlantic sturgeon (*Acipenser oxyrinchus oxyrinchus*) experienced severe declines due to habitat destruction and overfishing beginning in the late 19^th^ century. Subsequent to the boom and bust period of exploitation, there has been minimal fishing pressure and improving habitats. However, lack of recovery led to the 2012 listing of Atlantic sturgeon under the Endangered Species Act. Although habitats may be improving, the availability of high quality spawning habitat, essential for the survival and development of eggs and larvae may still be a limiting factor in the recovery of Atlantic sturgeon. To estimate adult Atlantic sturgeon spatial distributions during riverine occupancy in the Delaware River, we utilized a maximum entropy (MaxEnt) approach along with passive biotelemetry during the likely spawning season. We found that substrate composition and distance from the salt front significantly influenced the locations of adult Atlantic sturgeon in the Delaware River. To broaden the scope of this study we projected our model onto four scenarios depicting varying locations of the salt front in the Delaware River: the contemporary location of the salt front during the likely spawning season, the location of the salt front during the historic fishery in the late 19^th^ century, an estimated shift in the salt front by the year 2100 due to climate change, and an extreme drought scenario, similar to that which occurred in the 1960’s. The movement of the salt front upstream as a result of dredging and climate change likely eliminated historic spawning habitats and currently threatens areas where Atlantic sturgeon spawning may be taking place. Identifying where suitable spawning substrate and water chemistry intersect with the likely occurrence of adult Atlantic sturgeon in the Delaware River highlights essential spawning habitats, enhancing recovery prospects for this imperiled species.

## Introduction

The Delaware River Estuary historically supported the largest spawning population of Atlantic sturgeon (*Acipenser oxyrinchus oxyrinchus*) until over-fishing and habitat degradation caused sharp declines in their numbers [1]. The Delaware River Atlantic sturgeon fishery was short lived; peak landings of 2,900 mt occurred in 1888 and dropped more than 90% by the turn of the century [2]. In addition to over-fishing, navigation projects in the Delaware River Estuary resulted in the removal of over 1.5 x 10^8^ m^3^ of sediments, causing major changes in substrates, tidal flows, and salinity [3], [4], which likely altered Atlantic sturgeon spawning habitats. Recently the New York Bight Distinct Population Segment (DPS) of Atlantic sturgeon, which includes individuals from the Delaware River, was listed as endangered [5]. Although directed harvest of Atlantic sturgeon ended in 1998 [6], the results of historic overharvest coupled with habitat change and ongoing issues of bycatch mortality have resulted in a > 99% decline from historic abundance levels to < 300 spawning adults annually [7]. 

Like all sturgeons (family *Acipenseridae*), Atlantic sturgeon require flowing freshwater and adherence of eggs to appropriate substrate for successful spawning [8]. Eggs and larvae of sturgeons are salt intolerant and require salinity levels below 0.25 PSU to ensure survival [9]. Suitable spawning substrates vary from coarse sands, hardpan clay, to bedrock [10], [11]. In contrast, unconsolidated fine grain materials will adhere to the developing embryo resulting in abnormal development or mortality [11]. 

In the Delaware River, information on the location and timing of Atlantic sturgeon spawning in relation to salinity is limited, although it was believed to occur in a region ± 32 km bracketing the freshwater/saltwater interface [12]. These observations are based on fishery dependent data collected from Atlantic sturgeon landed for caviar during the late 19^th^ and early 20^th^ centuries [2] and are not consistent with recent findings on spawning requirements in other rivers [9]. Although these historic location observations may include both spawning and non-spawning adults, they still provide insights into the habitat usage of Atlantic sturgeon in the Delaware River. Historic accounts suggest that Atlantic sturgeon primarily utilized the area between Bombay Hook, DE (river kilometer (rkm) 61) and Chester, PA (rkm 130), and occasionally moved further upriver after the peak of the season in late-May [12]. 

Modifications made to the Delaware River over the last century have altered many historic Atlantic sturgeon spawning areas by changing the location of the salt front and composition of substrates [3], [4]. In light of the recent decision to list the NY Bight DPS as endangered, locating Atlantic sturgeon spawning areas is a high priority research need for the conservation and recovery of this imperiled species [5], especially given the renewed channel-deepening efforts in the Delaware River by the Untied States Army Corps of Engineers [13]. 

Unfortunately, the diminished population of spawning Atlantic sturgeon in the Delaware River (< 300 adults) [7] creates an impediment in identifying essential habitats. To overcome this hurdle, we coupled multiple years of acoustic biotelemetry detections with a presence-only modeling technique to determine if the salt front location and sediment composition are significant predictors of adult Atlantic sturgeon locations in the Delaware River during the spawning season. We projected our Atlantic sturgeon distribution model onto a series of historic and future Delaware River flow scenarios to approximate the likely location and distribution of suitable habitat available under each scenario. The ability to forecast the locations of Atlantic sturgeon and the distribution of suitable habitat given a changing climate provides insights into what the future may hold for a population showing sparks of recovery. Our analysis is a significant step in developing a quantitative spatial framework for managing this endangered species in an urbanized river. 

## Methods

### Ethics Statement

Capture and handling of Atlantic sturgeon was authorized under NMFS Permit No. 16507-01 and transmitter implantations were performed under MS-222 (tricaine methanesulfonate) to minimize stress and injury. Prior to any sampling, all actions were approved by the Delaware State University Institutional Animal Care and Use Committee. Daily detection data are available through correspondence with D. Fox. 

### Study Area

The study area focused on the tidal portion of the Delaware River from the confluence with the Chesapeake and Delaware Canal (C&D Canal; rkm 94) to the head of the tide at Trenton, NJ (rkm 210). The Delaware River is the largest undammed river in the Eastern United States and flows 530 km from New York to the mouth of the Delaware Bay. It contains the worlds largest freshwater, and one of the United States largest port complexes hosting approximately 3,000 deep-draft vessels per year [14]. The Delaware River Basin encompasses Delaware, New Jersey, New York, and Pennsylvania and covers over 35,000 km^2^, providing drinking water to over 5% of the United States population. 

The main freshwater input into the estuary comes from the Delaware River with a mean flow of 330 m^3^s^-1^ measured at Trenton, NJ [4]. Since 1877, the removal of 3.7 x 10^8^ m^3^ of sediment through dredging increased the mean channel depth of the tidal Delaware River (rkm 94-210) from 6.1 to 12.2 m, increasing tidal amplitude at the head of the tide (rkm 210) by nearly two-fold from 1.3 to 2.5 m [4]. 

### Location Data

 Atlantic sturgeon locations were estimated using passive acoustic biotelemetry. During April and May of 2009-2012, a total of 195 adult Atlantic sturgeon were implanted with long-lived acoustic transmitters (VEMCO V-16, 6-H, ~ 6.4 year battery life, 90 s mean transmission interval) 3-15 km off the Delaware coast, using protocols developed previously for Gulf sturgeon (*A. o. desotoi*) [15]. VEMCO VR-2W receivers were utilized to detect telemetered individuals when in range ( > 80% detection probability at distances of 1 km) [16]. Daily modal locations of individual Atlantic sturgeon were defined as the receiver location, which had the greatest number of hours with detections for that individual in a given day. In the rare occurrence of a daily mode occurring at multiple locations in a given day, daily modal location was assigned randomly to one of the locations with the highest number of hours. Atlantic sturgeon are capable of transiting the study area on a daily basis [16]; therefore, we treated daily modal locations as independent observations.

### Substrate composition

**Figure 1:**
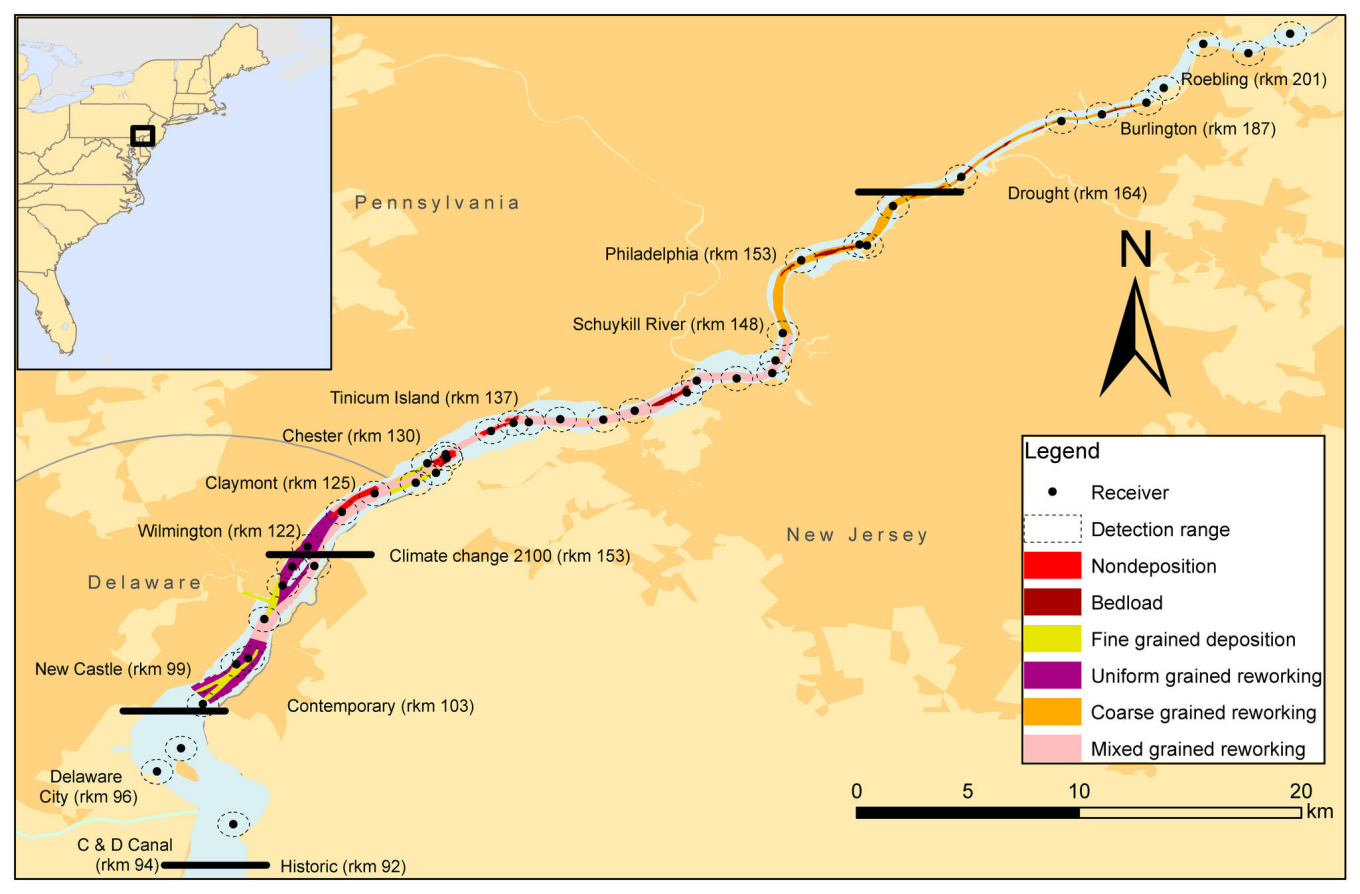
Acoustic receiver locations and 1 km detection radius overlain on the sediment composition map of the Delaware River (adapted from Sommerfield and Madson 2003), location of the salt front shown given four different scenarios, urban areas indicated through dark shading.

 Substrate composition was provided by a hydrographic study of the Delaware Estuary conducted from 2001-2002 by the University of Delaware [17], which quantified substrate at depths > 6 m in the Delaware River at a horizontal resolution of 1 m. This survey identified six sediment types: fine deposition (mud and fluid mud), bedload (moderately well sorted sand and gravel), fine reworking (mud), mixed reworking (mixed gravel, sand, and mud), coarse reworking (poorly sorted sand and gravel), and nondepositional (cobble and bedrock) (Figure 1) [17]. Atlantic sturgeon occurrence in the Delaware River corresponds with the deep-water habitat evaluated by the hydrographic survey [16]. Therefore, passive acoustic receiver locations were assigned a substrate type that covered the majority of the area within 1 km of the receiver location, which has previously been identified as the detection range of receivers in this environment [16] (Figure 1). Substrate composition in the Delaware River has changed since the end of the 19^th^ century [4], unfortunately no known records of historic sediments exist; therefore, substrate composition found in the contemporary hydrographic survey [17] was used for the projection of all model scenarios. 

To determine if the distribution of sediment type utilized by Atlantic sturgeon was significantly different than the distribution of sediment occupied by the receivers in the Delaware River we used a Chi-squared goodness-of-fit test [18]. Statistical significance was determined at *P* < 0.05 for all analyses.

### Location of the Salt Front

 The daily location of the salt front in the Delaware River was provided by the Delaware River Basin Commission (DRBC), and was based on the rolling seven-day average location of the 250 mg/L chloride concentration. We computed the distance between daily location of the salt front and daily modal Atlantic sturgeon locations for every day a given individual was in the Delaware River. 

### Presence-only Modeling

Recent advances in species distribution modeling have utilized presence-only records to estimate the probability of a species occurrence [19]. The MaxEnt program (MaxEnt v3.3.3) has been used to mediate the need for presence-absence data by relying solely on presence-only records along with associated environmental and geographical attributes [20]. MaxEnt starts with the maximum entropy of a given area and then constrains that area using provided environmental characteristics associated with the species occurrence [20]. This creates a species distribution map of the probability of occurrence given the input environmental features, which may be considered a surrogate for habitat suitability modeling [20]. MaxEnt also generates a Receiver Operating Characteristics (ROC) curve, which estimates goodness of fit by calculating the Area Under the Curve (AUC). An AUC of 0.5 is considered the result of a random distribution (non-predictive model) while an AUC > 0.9 is considered to have outstanding discrimination [21]. A cross-validation resampling procedure can be utilized to determine out of sample estimates of performance and uncertainty in the model [19].

We used the MaxEnt approach to model Atlantic sturgeon distributions in the tidal Delaware River during the spawning season as provided by detections in the passive receiver array from 2009-2012. Daily modal location estimates for individual Atlantic sturgeon, with associated sediment type and distance from the salt front, were used with a ten-fold cross-validation procedure to train and test the MaxEnt model [22]. In the MaxEnt program options, the regularization value was set at one to avoid model over-fitting, convergence threshold was 10^-5^ and the maximum number of iterations was 500 [19,20]. Analysis of omission and ROC curves were examined to determine the function of the omission rate in terms of the cumulative threshold and sensitivity given the fractional predicted area respectively [20]. The AUC was calculated for the ROC to determine the models ability to discriminate between its prediction and random occurrences. 

Once the model was developed, we projected the estimated probability of adult Atlantic sturgeon occurrence onto the contemporary conditions of the tidal Delaware River. Additionally, we determined the importance of covariates in the model by computing the percent permutation importance to the performance of the model by each covariate. Importance values were calculated by random permutation of the covariates independently and measuring the resulting decrease in the AUC [20]. Covariate importance was also estimated with jackknife tests where the model was run with the exclusion and isolation of each covariate while recording the resulting changes in performance [20]. 

To determine the effect of a migrating salt front on the Delaware River, we projected the developed model onto three additional scenarios of the location of the salt front. The first scenario was the historical location of the salt front during the peak of the sturgeon fishery in the late 19^th^ century [12], as estimated during the fishing season (rkm 92). Second, we used an 11 km upstream shift (rkm 114) of the salt front in 2100 resulting from sea level rise as modeled by the Environmental Protection Agency [23] (Figure 1). The final scenario used the location of the salt front given the extreme drought conditions that were observed in the early 1960’s and represent the highest upstream records of the salt front (rkm 164) [23]. 

## Results

Forty-six receivers were deployed and maintained in the tidal Delaware River from 2009-2012. The mean distance between receiver locations was 2.5 km enabling near continuous detection of telemetered individuals with individuals being detected at least 20 hours of every day while in the study area. Thirty-six of the 46 receiver locations fell within the confines of the recent hydrographic survey of the sediments (Figure 1). The majority of receiver locations were associated with mixed-grained reworking substrate (44%) followed by fine-grained reworking (17%), coarse-grained bedload (17%), nondepositional (17%), coarse-grained reworking (8%) and fine-grained deposition (3%). The ten receivers that fell outside the confines of the hydrographic survey were utilized for location information (i.e. distance to salt front) only and not substrate type. 

**Figure 2:**
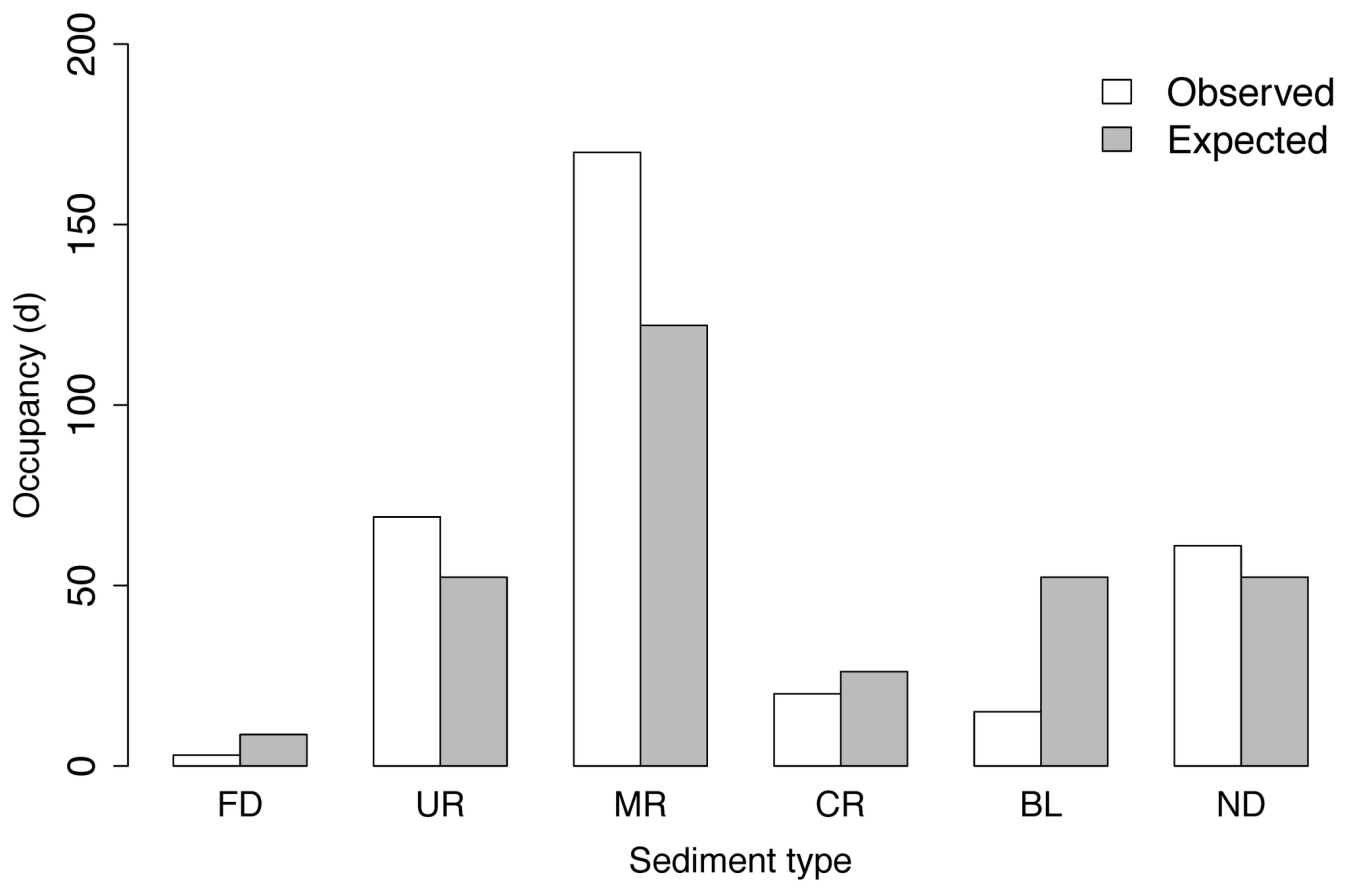
Number of days adult Atlantic sturgeon were observed occupying each substrate type with expected number of days by each sediment type: FD- Fine Deposition, UR- Uniform Reworking, MR- Mixed Reworking, CR- Coarse Reworking, BL- Bedload, ND- Nondepositional.

Of the 195 adult Atlantic sturgeon telemetered, 12 individuals made a total of 20 likely spawning migrations from the Atlantic Ocean to the Delaware River between 2009-2012 (Table 1). Atlantic sturgeon remained in the Delaware River for 7-70 d in April-July, and traveled as far upstream as Roebling, NJ (rkm 201), occupying sediment types at a proportion that was significantly different from the distribution of available sediment types. Atlantic sturgeon selected for substrates consisting of mixed and uniform-grained reworking material (χ^2^ = 63.9, *df* = 5, *P* < 0.0001) (Figure 2). 

**Table 1:** Individual adult Atlantic sturgeon that entered the Delaware River from 2009-2012 during the likely period of spawning with sex, length, weight, timing of occupancy, and maximum river kilometer.

ID	Capture date	Sex	Fork length (cm)	Weight (kg)	Date of arrival	Date of departure	Days of occupancy	Max rkm*
1225	4/27/2006	Male	158	n/a	5/11/2009	n/a	n/a	141
2433	4/5/2009	Male	178	57	5/14/2009	5/30/2009	16	125
2442	4/8/2009	Male	166	50	5/5/2009	6/15/2009	41	129
2433	4/5/2009	Male	178	57	4/27/2010	5/19/2010	22	130
2442	4/8/2009	Male	166	50	5/7/2010	6/4/2010	28	129
4954	4/9/2010	Male	154	41	4/15/2010	5/2/2010	17	115
4965	4/20/2010	Male	171	53	5/8/2010	6/6/2010	29	130
2433	4/5/2009	Male	178	57	4/29/2011	5/29/2011	30	135
2442	4/8/2009	Male	166	50	5/1/2011	5/28/2011	27	122
4954	4/9/2010	Male	154	41	4/14/2011	4/17/2011	3	92
4956	4/12/2010	Male	184	58	5/8/2011	6/8/2011	31	201
4958	4/12/2010	Female	196	79	5/7/2011	5/23/2011	16	135
4965	4/20/2010	Male	171	53	5/13/2011	5/30/2011	17	190
4980	4/22/2010	Female	206	84	5/21/2011	7/30/2011	70	148
2433	4/5/2009	Male	178	57	4/22/2012	5/23/2012	32	129
2442	4/8/2009	Male	166	50	4/17/2012	6/6/2012	50	135
2465	4/3/2012	Male	180	63	5/6/2012	5/25/2012	20	120
2466	4/3/2012	Male	167	42	5/13/2012	6/22/2012	39	176
2488	4/9/2012	Female	208	98	5/9/2012	5/29/2012	10	130
2493	4/12/2012	Male	163	43	5/6/2012	6/1/2012	26	135

* rkm – River Kilometer

**Figure 3:**
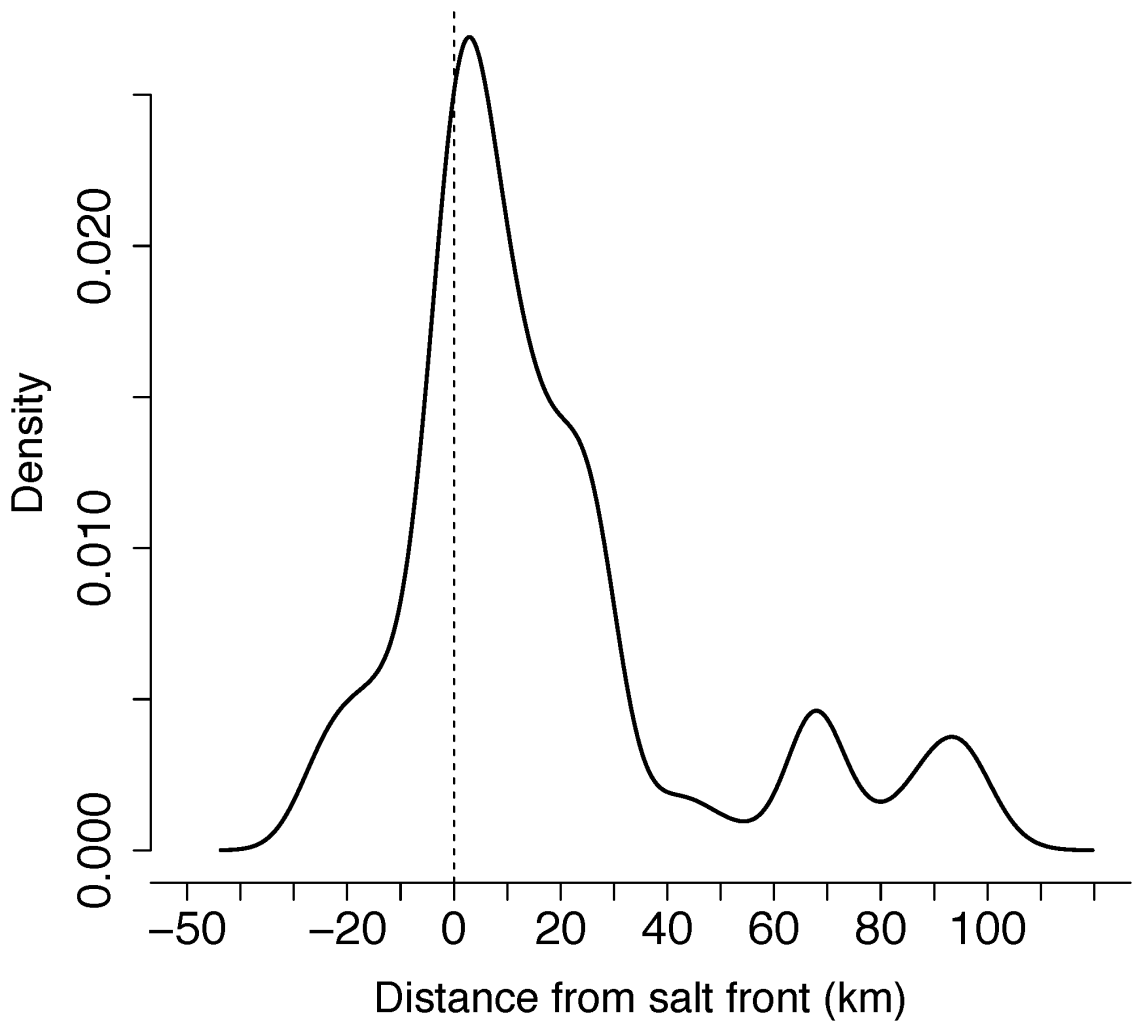
Density of observations by distance from salt front for telemetered adult Atlantic sturgeon in the Delaware River during the likely period of spawning from 2009-2012.

The average location of the salt front during adult Atlantic sturgeon occupancy of the Delaware River during this study ranged from rkm 92 (2011) to rkm 112 (2009 and 2012). Atlantic sturgeon inhabited areas of the river ± 30 km from the estimated salt front for 84% of the time with smaller peaks occurring 60-100 km above the salt front for 16% of the time (Figure 3). 

**Figure 4:**
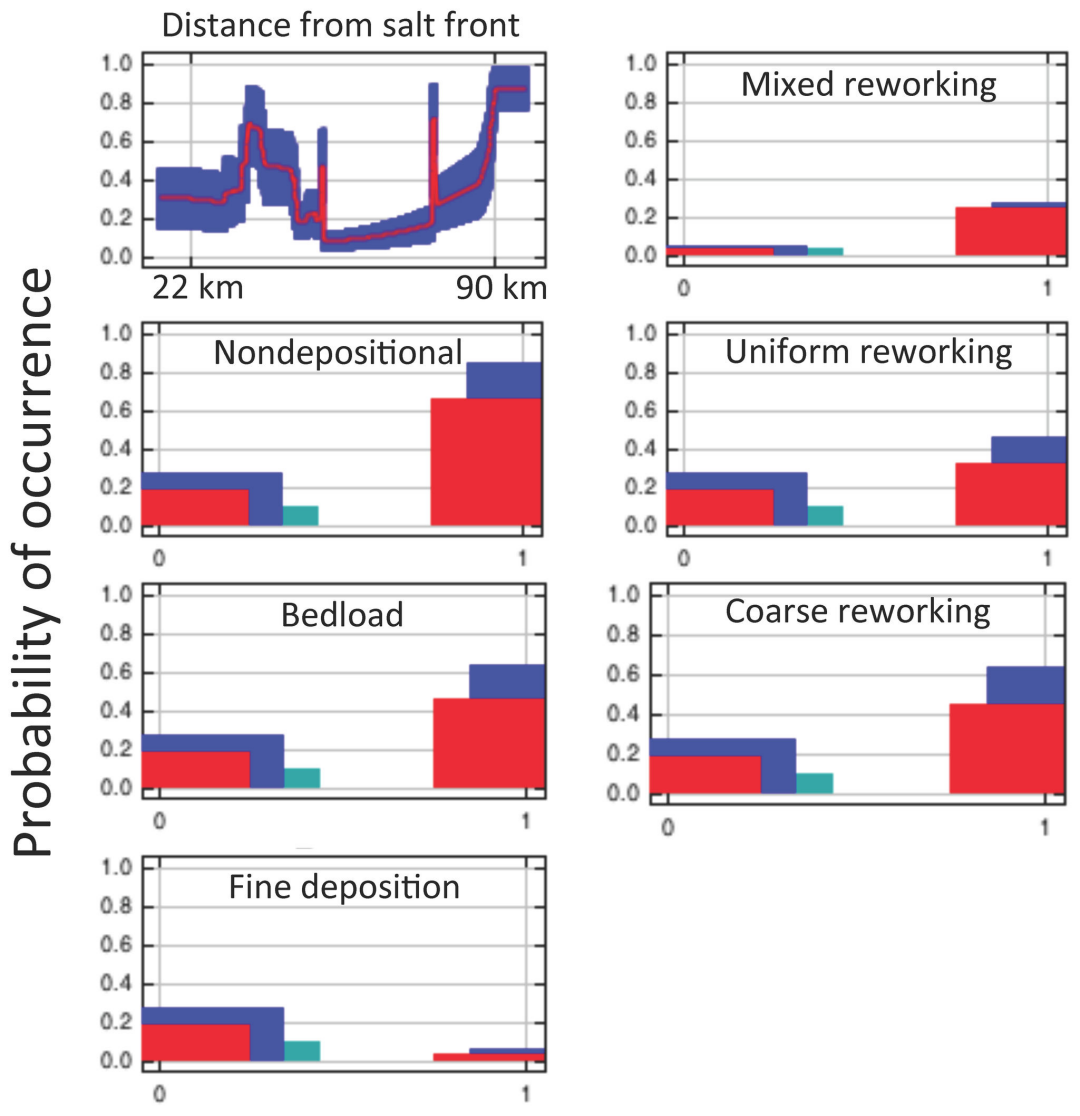
Contemporary MaxEnt mean response curves of the covariates distance to the salt front (km), mixed reworking (mixed gravel, sand, and mud), nondepositional (cobble and bedrock), uniform reworking (mud), bedload (moderately well sorted sand and gravel), coarse grained reworking (poorly sorted sand and gravel), and fine deposition (mud and fluid mud). (0 = absence of covariate, 1 = presence of covariate). Mean is in red with ± one standard deviation in blue (two shades for the categorical substrate covariates).

 Our MaxEnt model depicted the distribution of Atlantic sturgeon by incorporating the passive acoustic detections of telemetered individuals along with associated substrates and distances to the salt front. The contemporary model estimated Atlantic sturgeon distributions very well with an AUC of 0.900 with the relative location of the salt front providing the greatest contribution (43.2%) to the model. The response curve for the covariate of relative location of the salt front (all other covariates held at average value) resembles the distribution of occurrence histogram with noticeably strong peaks at 65 and 90 km above the salt front (Figure 4). Mixed-grained reworking sediments (26.3%), and nondepositional substrates (18.9%) where the next highest contributors, while the remaining four substrate types combined contributed < 13% to the model performance (Table 2). Permutation importance is in agreement with the jackknife plots (Figure S1). Evaluation of the response curves for the six sediments types reveal a higher probability of occurrence in areas with nondepositional, bedload, and coarse reworking substrates, while areas with fine deposition substrate had a lower probability of occurrence (Figure 4). 

**Table 2:** Model and covariate evaluation for the contemporary analysis and three projected scenarios of salt front location: historical location (rkm 92), location given climate change (rkm 114), and location given drought conditions (rkm 164).

	Location of Salt Front (RKM)			
	Contemporary (103)	Historic (92)	Climate Change (114)	Drought (164)
Average AUC	0.89	0.89	0.90	0.90
Distance from Salt Front Importance	43.2	44.7	40.4	42.2
Mixed Reworking Importance	26.3	25.4	28.4	25.6
Nondepositional Importance	18.9	19.5	19.9	20.3
Uniform Reworking Importance	3.8	3.5	4.0	3.9
Bedload Importance	2.1	2.0	2.1	2.2
Fine Deposition Importance	1.5	1.5	1.2	1.7
Coarse Reworking Importance	4.2	3.4	4.1	4.1

When the model was projected on to the three salt front location scenarios, the model continued to preform at a high level with AUCs of 0.893 for the historic location of the salt front, 0.895 given climate change, and 0.898 under the extreme drought scenario (Table 2). Additionally, the permutation importance of the salt front location and substrate distributions varied less than ±3% for all variables in the three projected scenarios (Table 2). Response curves for the three additional scenarios were similar to the response curves for the contemporary model (Figure S2). MaxEnt ROC and omission rate plots depicting the fraction of background habitat versus the cumulative threshold of suitable habitat for the contemporary analysis and three scenarios are available in Figure S3.

**Figure 5:**
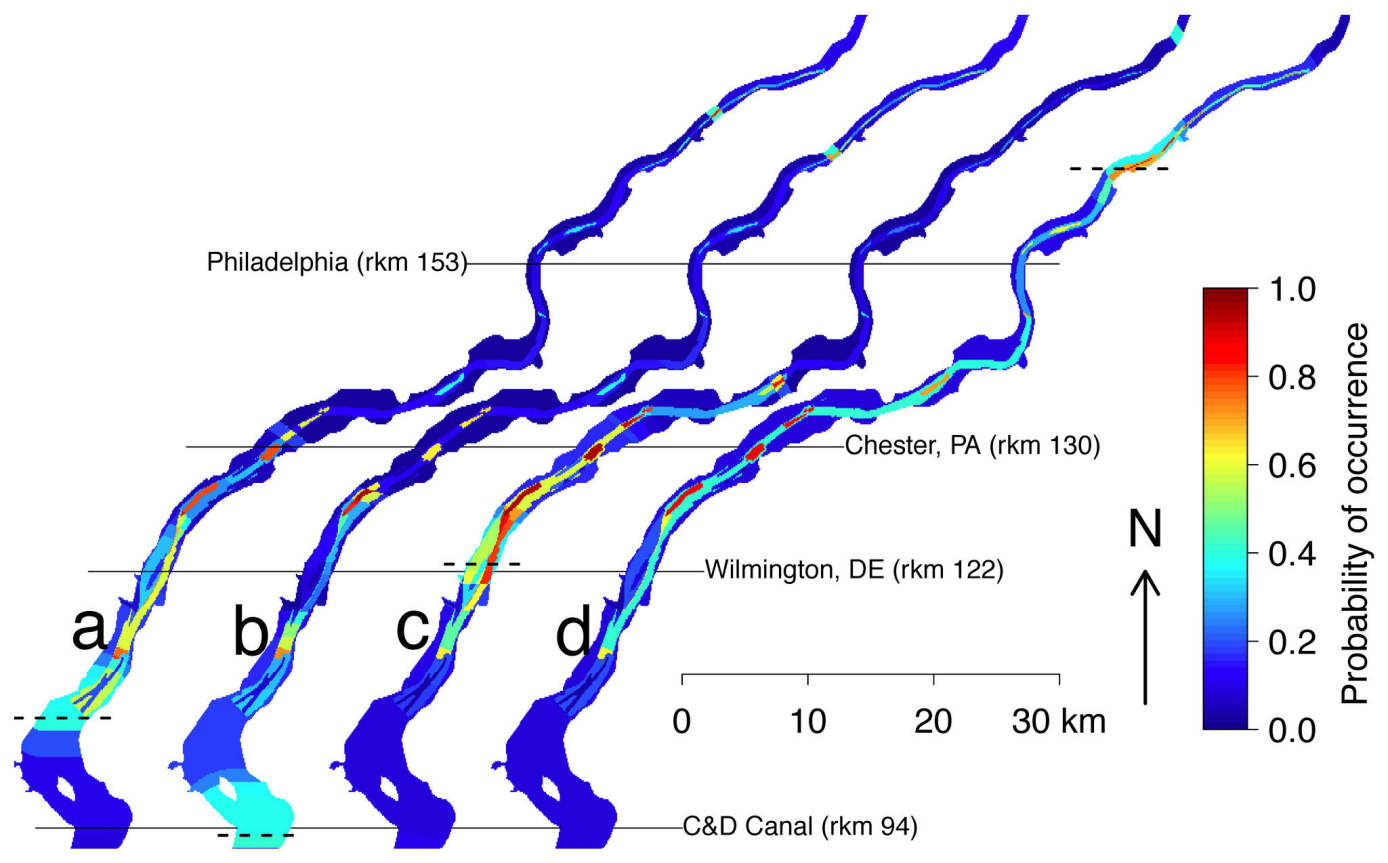
Projected areas of adult Atlantic sturgeon occurrence for 4 different salt front location scenarios: a- contemporary location (rkm 103), b-historical location (rkm 92), c- location given climate change (rkm 114), d- location given drought conditions (rkm 164).

 The contemporary probability distribution map (Figure 5a) suggests that Atlantic sturgeon occupy the region from New Castle, DE (rkm 99) to Tinicum Island, PA (rkm 137), with high concentration areas near Claymont, DE (rkm 125) and Chester, PA (rkm 130). These high concentration areas contain coarse-grained and nondepositional bedrock habitat suitable for spawning (Figure 1). Additionally, there were signals of occupancy as far upstream as Burlington, NJ (rkm 187; Figure 5a). 

Our estimates, given the historic location of salt front (rkm 92), suggest adult Atlantic sturgeon distributions were concentrated over a wide area (~35 km) extending from downstream of Delaware City (rkm 96) to Chester, PA (rkm 130) with a low probability of occurrence in the reaches from Chester, PA (rkm 130) to Burlington, NJ (rkm 187; Figure 5b). The likely distribution of adult Atlantic sturgeon under the scenario of increased seawater intrusion due to climate change and sea level rise is constrained and shifted upstream. Under this scenario, Atlantic sturgeon would principally occur from Wilmington, DE (rkm 122) to Tinicum Island, PA (rkm 137) with the highest concentrations near Claymont, DE (rkm 125) and Chester, PA (rkm 130; Figure 5c). Under extreme historic drought conditions, the distribution of adult Atlantic sturgeon was constricted to several localized spots occurring near Claymont, DE (rkm 125), Chester, PA (rkm 130), and in the waters adjacent to Philadelphia (rkm 153; Figure 5d).

## Discussion

Historic overfishing followed by habitat degradation of riverine environments essential to Atlantic sturgeon spawning and rearing has depleted their populations and stalled recovery. The lack of population increase given improved conditions in the Delaware River and the absence of directed fishing pressure were listed as the main drivers for the recent listing of the Atlantic sturgeon NY Bight distinct population segment under the Endangered Species Act [5]. We used acoustic telemetry, sediment information, and location of the salt front to determine if these factors play a significant role in the occurrence of adult Atlantic sturgeon during the spawning season in the Delaware River. Additionally, we were able to project the impact of sediment type and salt front location onto historic, contemporary, and future scenarios in the region to determine how these environmental predictors might mediate the distribution of adult Atlantic sturgeon in the Delaware River. 

Limited knowledge of riverine requirements by adult Atlantic sturgeon [7] coupled with their current conservation status [5] underscores the need for an improved understanding of the drivers that dictate habitats utilized during spawning migrations. While in the riverine environment, Atlantic sturgeon require both staging and spawning habitats, occupying the staging habitats for the majority of the time, making several short, directed spawning runs of 10-50 km to and from spawning habitats during the spawning season [24]. The use of MaxEnt coupled with passive telemetry has enabled us to identify important stretches of the Delaware River for adult Atlantic sturgeon, providing an alternative to intense, in-river sampling in a heavily trafficked urban river. 

 Adult Atlantic sturgeon in the Delaware River showed an affinity for areas near the salt front. The majority of time was spent ~ 30 km downstream to 30 km upstream of the salt front, typically between New Castle, DE (rkm 99) and the Schuylkill River, PA (rkm 148). With adjustments taken for the migration of the salt front over the last century, our findings mirror accounts from the 1880’s: “From this point southward 20 miles, and northward as many more, it is probable that a large part of the spawning [*of Atlantic sturgeon*] now occurs” [12]. This account references the location of the salt front and believed spawning to take place ± 32 km from this point [12]. Although, we now know that Atlantic sturgeon are incapable of successfully reproducing in areas downstream of the salt front [9] this historic account alludes to important habitats in close proximity to the salt front. 

Our modeling efforts suggest adult Atlantic sturgeon locations within the Delaware River are influenced by substrate composition and the location of the salt front. While the majority of Atlantic sturgeon acoustic detections were within 30 km of the salt front, the response curves show additional high probability of occurrence around 65 km above the salt front and then again at areas > 90 km above the salt front. While occurrences well upstream of the salt front were observed in the telemetry data, the high contribution to the model above 90 km is likely an artifact of lack of substrate data at the upper end of the study area. Atlantic sturgeon appear to select for areas with coarse reworking and nondepositional substrates, which corresponds with previous findings for juvenile Atlantic sturgeon in the Delaware River [16], adults and late stage juveniles in the Hudson River [25], and is a common theme throughout the range of Atlantic sturgeon [26],[27]. 

 By relating the locations of various substrates to the distance from the salt front, we were able to model distributions of Atlantic sturgeon and highlight key areas of congregation based on these features. Our model estimates a distribution bracketing the salt front and indicates two areas of high concentration occurring upstream of the salt front (Figure 5a). These two areas near Claymont, DE (rkm 125) and Chester, PA (rkm 130) are largely composed of bedrock habitat that is ideal for spawning [11]. The high probability of Atlantic sturgeon occupancy in these two relatively small areas and the presence of suitable spawning habitat provide strong evidence that spawning is taking place in these reaches. Without the collection of eggs or larvae we cannot confirm spawning activity in these areas; however, young-of-the-year Atlantic sturgeon have been collected in close proximity suggesting nearby spawning [28]. 

By projecting the model onto historic conditions, we estimated the probability of occurrence when Atlantic sturgeon harvests were at their peak in the late 19^th^ century (Figure 5b) [12]. These historic conditions, with the salt front depressed further downstream, allowed adult Atlantic sturgeon to utilize large expanses of suitable habitat during the spawning season. The probability of occurrence map for historic conditions showed a reduction in Atlantic sturgeon in the lower extent of our study area compared with the contemporary model. This reduction is likely a combination of lack of available substrate data for that stretch of the Delaware River as well as a shift in the range of adult Atlantic sturgeon downstream of the area covered by this study and not a true lack of occurrence during that time period. In contrast, increased salt-water intrusion from the marine environment that is likely to occur under predicted scenarios of climate change and sea level rise [23], markedly constrain areas of suitable habitat for Atlantic sturgeon in the Delaware River (Figure 5c). The increased seawater inundation of the Delaware River due to sea level rise will soon threaten the two small reaches this study has highlighted as potential spawning areas. 

Under projected climate change by 2100, an 11 km upstream shift in the salt front is predicted as a direct result of sea level rise [23]. This compression of the freshwater portion of the Delaware River is further exacerbated by the ongoing Delaware River Main Channel Deepening Project [13], which upon completion will increase channel depths from 12.2 to 13.7 m. This depth increase is expected to shift the salt front an additional 4 km upstream [4] for a total shift of 15 km. Additionally, the expansion of the Panama Canal, allowing vessels with a draft of 15 m compared to the current maximum draft of 12 m, will increase the demand for deeper draft East Coast ports and may increase pressure to deepen the Delaware River beyond 13.7 m [29]. Further deepening of the Delaware River will likely result in increased inundation and further restriction of suitable habitat for Atlantic sturgeon. 

Compounding the effect of increased salinity from seawater inundation is the upstream movement of the turbidity maximum zone, which is directly linked to the location of the salt front in the Delaware River [30]. The upstream shift of the turbidity maximum zone will likely increase sedimentation rates [30] in these highlighted reaches and transform the existing hard-bottom substrates into areas of fine deposition substrate in which our model has shown the probability of occurrence of adult Atlantic sturgeon is low. 

In addition to severely reducing available staging and spawning habitats, upstream movement of the salt front also concentrates Atlantic sturgeon in areas of the river with the highest volume of commercial traffic. This overlap of an endangered species that is vulnerable to vessel strikes [28], [31] [32], with deep draft vessels could result in a situation that hinders recovery of Atlantic sturgeon in the Delaware River. 

The characterization of adult Atlantic sturgeon riverine habitat has proven notoriously difficult, mainly because of their large size and occupancy of the main channels of large river systems, which often directly overlap with commercial shipping activity. With the aid of biotelemetry and newly developed species distribution models, which utilize presence-only records, we were able to estimate the occupancy of adult Atlantic sturgeon during spawning migrations, a critical stage in their life history. Furthermore, the convergence of model outputs with historic information on Atlantic sturgeon distributions not only provides support for our model, but also suggests that this approach is a valuable hind-casting tool. Even though the exact geographic locations from the historic fishery are vague, the description of habitats was very similar to habitats that were estimated through our modeling efforts. While the Delaware River has undergone large alterations since the peak of the Atlantic sturgeon fishery in the late 19^th^ century [4], the habitat requirements for Atlantic sturgeon have remained constant, resulting in a reduction in available staging and spawning habitat. Additionally, we were able to employ our model to forecast the distributions of Atlantic sturgeon given alterations to the location of the salt front in the Delaware River. These forecasts provide insights into the challenges we will face as we struggle to conserve and recover this imperiled species while balancing the economic needs of the mid-Atlantic region. 

## Supporting Information

Figure S1 **Jackknife plots of the test gain of the covariates for the contemporary analysis as well as the three additional scenarios of the location of the salt front.**
(TIFF)

Figure S2 **Mean response curves for the MaxEnt analysis of the three scenarios for the covariates distance to the salt front (km), mixed reworking (mixed gravel, sand, and mud), nondepositional (cobble and bedrock), uniform reworking (mud), bedload (moderately well sorted sand and gravel), coarse grained reworking (poorly sorted sand and gravel), and fine deposition (mud and fluid mud).** (0 = absence of covariate, 1 = presence of covariate). Mean is in red with ± one standard deviation in blue (two shades for the categorical substrate covariates.
(TIFF)

Figure S3 **MaxEnt receiver operating curves and plots of the omission rate for test model runs of the contemporary location of the salt front as well as the three different scenarios of the location of the salt front.**
(TIFF)
