# Supplementary figures and images for: Shifting Distributions of Adult Atlantic Sturgeon Amidst Post-Industrialization and Future Impacts in the Delaware River: a Maximum Entropy Approach
Source: PLoS One. 2013 Nov 8;8(11):e81321. doi: 10.1371/journal.pone.0081321 (PMC3832663; doi:10.1371/journal.pone.0081321)

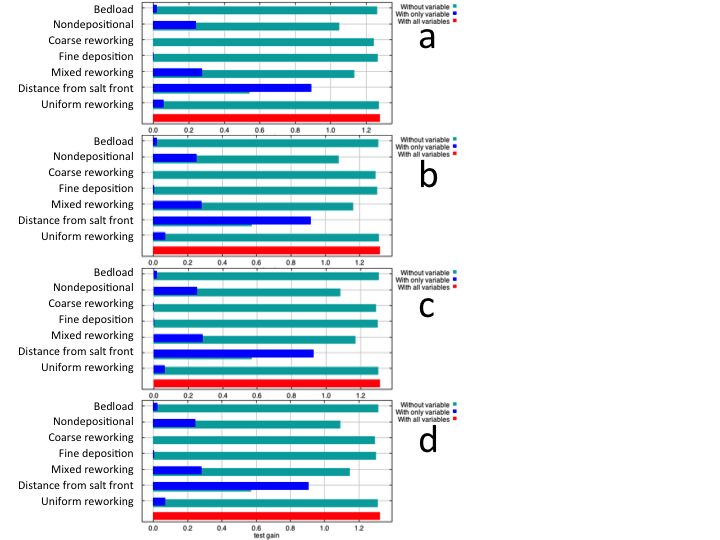

Supplement: Figure S1 — Jackknife plots of the test gain of the covariates for the contemporary analysis as well as the three additional scenarios of the location of the salt front. (TIFF) [file pone.0081321.s001.tiff]

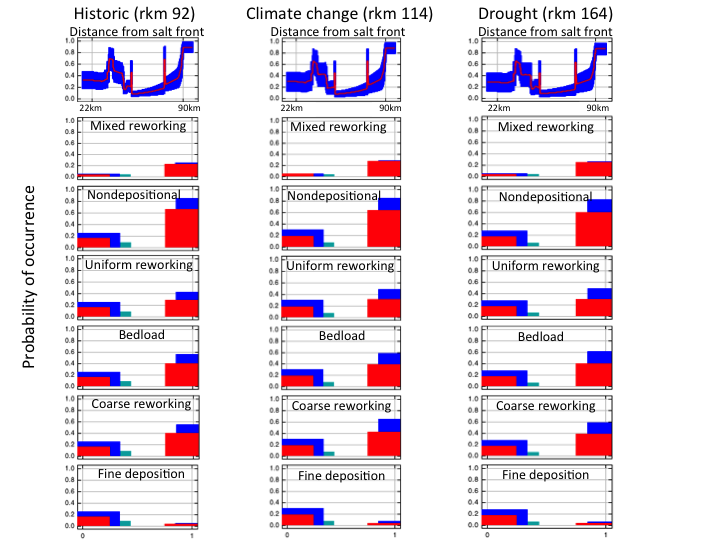

Supplement: Figure S2 — Mean response curves for the MaxEnt analysis of the three scenarios for the covariates distance to the salt front (km), mixed reworking (mixed gravel, sand, and mud), nondepositional (cobble and bedrock), uniform reworking (mud), bedload (moderately well sorted sand and gravel), coarse grained reworking (poorly sorted sand and gravel), and fine deposition (mud and fluid mud). (0 = absence of covariate, 1 = presence of covariate). Mean is in red with ± one standard deviation in blue (two shades for the categorical substrate covariates. (TIFF) [file pone.0081321.s002.tiff]

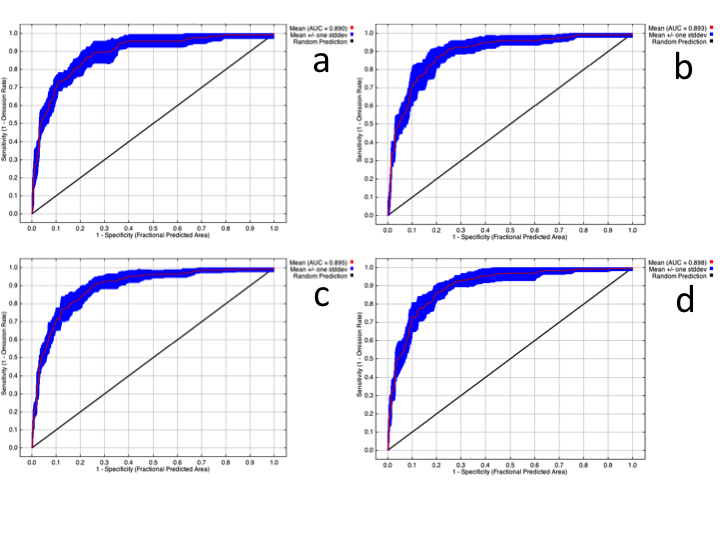

Supplement: Figure S3 — MaxEnt receiver operating curves and plots of the omission rate for test model runs of the contemporary location of the salt front as well as the three different scenarios of the location of the salt front. (TIFF) [file pone.0081321.s003.tiff]
